# Early Childhood Exposure to Endocrine Disrupting and Neurotoxic Chemicals: Associations with Internalizing and Externalizing Difficulties from Childhood to Adolescence in the Rhea Cohort, Crete, Greece

**DOI:** 10.3390/toxics13100854

**Published:** 2025-10-10

**Authors:** Chrysi Mouatsou, Katerina Margetaki, Mariza Kampouri, Marianna Karachaliou, Antonis Myridakis, Danae Costopoulou, Leondios Leondiadis, Euripides G. Stephanou, Lida Chatzi, Manolis Kogevinas, Katerina Koutra

**Affiliations:** 1 Department of Psychology, School of Social Sciences, University of Crete, 741 00 Rethymno, Crete, Greece; chrysamouatsou@gmail.com; 2 Clinic of Social Preventive Medicine, Department of Social Medicine, Faculty of Medicine, University of Crete, 700 13 Heraklion, Crete, Greece; katmargetaki@hotmail.com (K.M.); mar.kabouri@gmail.com (M.K.); marianna.karachaliou@isglobal.org (M.K.); 3 Institute of Environmental Medicine, Karolinska Institutet, 171 77 Stockholm, Sweden; 4 ISGlobal, Barcelona Institute for Global Health, 08036 Barcelona, Spain; 5 Centre for Pollution Research & Policy, Environmental Sciences, Brunel University London, Uxbridge UB8 3PH, UK; 6 Mass Spectrometry and Dioxin Analysis Laboratory, Institute of Nuclear & Radiological Sciences & Technology, Energy & Safety (INRASTES), National Centre for Scientific Research (NCSR) “Demokritos”, 153 10 Athens, Greece; costodan@rrp.demokritos.gr (D.C.); leondi@rrp.demokritos.gr (L.L.); 7 Department of Chemistry, University of Crete, 700 13 Heraklion, Crete, Greece; evris.stephanou@uoc.gr; 8 Department of Preventive Medicine, Keck School of Medicine, University of Southern California, Los Angeles, CA 90033, USA; chatzi@usc.edu

**Keywords:** organochlorine pesticides, polychlorinated biphenyls, phthalates, organophosphate pesticides, internalizing symptoms, externalizing symptoms, attention-deficit/hyperactivity disorder

## Abstract

Many common chemicals are known or suspected to harm brain development, and children are particularly vulnerable, yet research on their long-term effects on mental health is limited. This study investigated the associations of early childhood exposure to endocrine disrupting and neurotoxic chemicals with the development of internalizing, externalizing, and attention-deficit/hyperactivity disorder (ADHD) symptoms from early childhood through adolescence in 387 children from the Rhea cohort in Crete, Greece. At age 4, serum concentrations of 3 organochlorine pesticides and 14 polychlorinated biphenyls, and urinary concentrations of 7 phthalate metabolites and 6 dialkyl phosphate metabolites were measured. Children’s symptoms were assessed via maternal reports at ages 4, 6, 11 and 15 years. Using generalized estimating equation models, the study found that early exposure to hexachlorobenzene (HCB) and dichlorodiphenyldichloroethylene (DDE) was associated with increased externalizing symptoms across ages in girls [beta (95% CI): 0.20 (0.04, 0.37) and 0.11 (0.01, 0.21), respectively]. Among girls, low molecular weight (LMW) phthalates were also linked to elevated internalizing and externalizing symptoms, as well as ADHD-related difficulties [beta (95% CI): 0.15 (0.04, 0.26), 0.13 (0.01, 0.25), and 0.13 (0.02, 0.24), respectively]. Additionally, exposure to organophosphate pesticides was associated with increased externalizing and ADHD symptoms [beta (95% CI): 0.13 (0.04, 0.22) and 0.12 (0.04, 0.20), respectively]. The findings suggest that early childhood exposure to environmental chemicals may have long-term effects on emotional and behavioral development, with pronounced effects observed only in girls.

## 1. Introduction

Emotional and behavioral disorders, as well as neurodevelopmental disorders, such as Attention-Deficit/Hyperactivity Disorder (ADHD), are prevalent among children and adolescents [1,2]. These disorders are linked to adverse outcomes, including low academic performance [3], increased risk of substance use [4], and higher unemployment rates [5]. Furthermore, experiencing elevated symptoms during childhood and adolescence is associated with an increased likelihood of these disorders continuing into adulthood [6,7].

The etiology of these disorders is considered multifactorial and research has indicated potential connections of chemical exposure with neurodevelopmental impairments, behavioral deficits, and increased risk of ADHD [8,9,10]. Children are more susceptible to the effects of environmental chemicals than adults, making them particularly vulnerable to these adverse outcomes [11]. This heightened susceptibility is attributed to several factors, including the complexity of brain development [12] and the immaturity of detoxification mechanisms [13]. Furthermore, children have a higher relative exposure to environmental chemicals as they consume more food, water, and air relative to their body weight in comparison to adults [14]. Additionally, behaviors such as hand-to-mouth activities further elevate exposure [15]. Disruption of neurodevelopment due to environmental insults during this critical period can lead to irreversible and long-lasting effects, increasing the risk for developing various pathologies across the lifespan [16,17].

Research on postnatal exposure to persistent organic pollutants, such as organochlorine pesticides (OCPs) and polychlorinated biphenyls (PCBs), is less extensive than studies on fetal exposure, with most focusing on early postnatal periods. These studies have produced inconsistent results, particularly when examining the first 12 to 24 months of life [18,19,20,21], and findings on exposure later in childhood are also mixed. Some studies suggest associations between PCB exposure and increased emotional symptoms [22,23], while others report inverse associations [24]. Research on phthalates, compounds commonly found in personal care products and plastics [25], has shown positive associations with behavioral problems [26,27], teacher-reported ADHD symptoms [28], attention problems [29], and social difficulties [30], while a study on 7-year-olds from a Polish cohort found no link between exposure at age 2 and internalizing or externalizing problems [31]. Studies on organophosphate pesticides (OPs), primarily sourced from contaminated food, also show mixed results. While some studies link OP exposure to ADHD [32,33] and internalizing symptoms [34], others report null or even inverse associations with behavioral problems [24,35].

The biological mechanisms underlying the effects of these environmental chemicals on brain development are not fully understood, but both animal and human studies suggest that these effects may be sex-specific [36,37]. Most studies evaluating postnatal exposure to persistent organic pollutants have not considered sex as a key factor [21,22], while no significant sex differences have been reported by those that have investigated it [14,18,19]. In contrast, sex-specific effects have been more extensively examined in relation to phthalate and OP exposure, though the findings remain inconsistent. These studies show that the effects can differ between boys and girls depending on the specific chemical metabolite and the neurobehavioral domain assessed [26,29,34,38], while sex differences in the concentrations and metabolism of phthalates have been previously reported in the Rhea cohort [39,40].

Given the rising prevalence of emotional, behavioral and neurodevelopmental disorders [1,2], as well as their potential links to chemical exposures, a deeper understanding of the effects of chemical pollution on mental health is needed. Existing research is limited, often focusing on specific pollutants or developmental stages, with inconsistent findings on the role of sex. In this context, our study aims to investigate childhood exposure (at age 4) to chemicals, including OCPs, PCBs, phthalates, and organophosphate pesticides, in relation to the development of internalizing, externalizing, and ADHD symptoms from early childhood through adolescence (at ages 4, 6, 11, and 15 years), while also assessing potential sex-specific effects.

## 2. Materials and Methods

### 2.1. Study Population

The present study utilized data from the Rhea Study, an ongoing population-based mother-child cohort in Crete, Greece. A detailed description of the cohort is available in Chatzi et al. [41]. Briefly, pregnant women were initially approached in four clinics (both public and private) in the city of Heraklion between February 2007 and February 2008. To meet the eligibility criteria, women were required to reside in the prefecture of Heraklion, be over 16 years old, and have a good understanding of the Greek language. A total of 1610 pregnant women were recruited in the Rhea Study around the 12th gestational week, and 1363 singleton pregnancies were followed through delivery. Assessments were conducted twice during pregnancy and at birth admission. Children were evaluated at multiple developmental stages: infancy (approximately 9 and 18 months), early childhood (median 4.2 years), mid-childhood (median 6.5 years), pre-adolescence (around 11 years), and adolescence (15 years).

**Figure 1:**
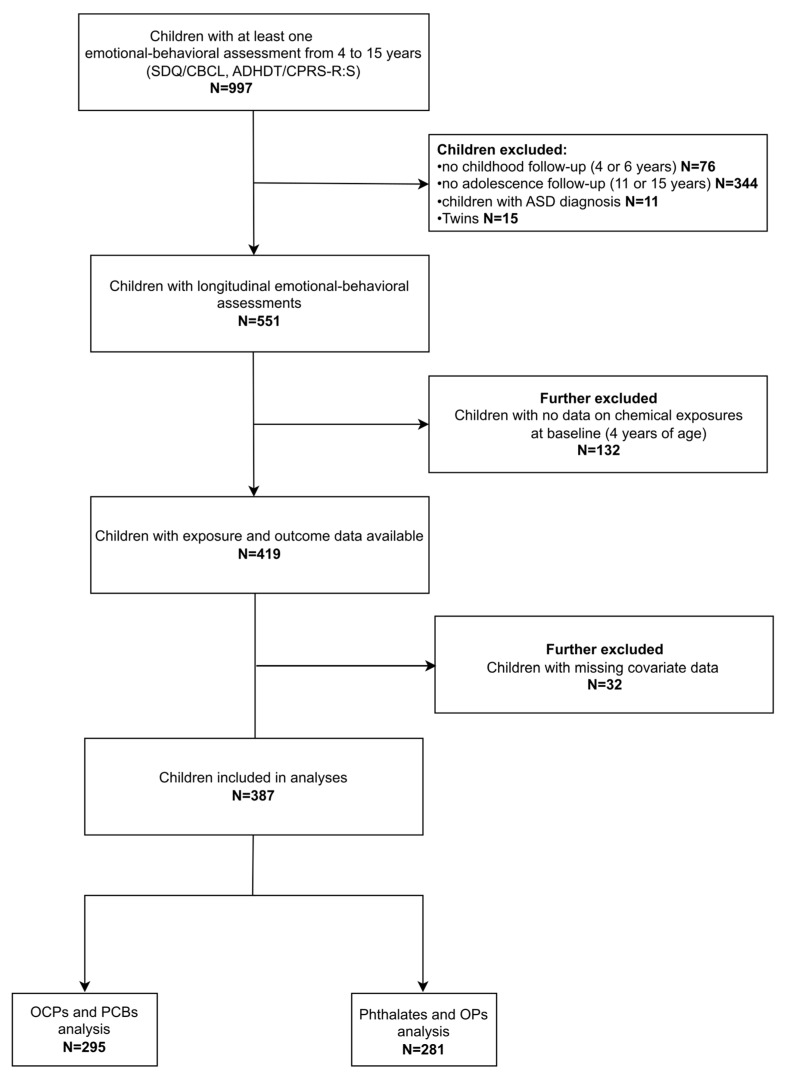
Flow diagram of the study population. Abbreviations: ADHDT: Attention Deficit Hyperactivity Disorder Test; ASD: Autism Spectrum Disorder; CBCL: Child Behavior Checklist; CPRS-R:S: Conners’ Parent Rating Scale-Revised: Short Form; OCPs: Organochlorine pesticides; OPs: Organophosphate pesticides; PCBs: Polychlorinated biphenyls; SDQ; Strengths and Difficulties Questionnaire.

For the purposes of this research, data from the assessments conducted at ages 4, 6, 11, and 15 years were utilized. In total, 997 children had available data on internalizing, externalizing, or ADHD symptoms for at least one of the four assessments. To capture development across both childhood and adolescence, we set the requirement that each child should have been assessed at least once at ages 4 or 6 and once at ages 11 or 15. Twins (N = 15) and children diagnosed with autism spectrum disorder (N = 11) were not included in the analyses. Data on chemical exposure at the beginning of the trajectory (4 years) were not available for 132 children. Further, 32 children (7.6%) were excluded due to missing covariate information. The final sample consisted of 387 children (Figure 1).

### 2.2. Exposure Assessment

Blood and urine samples were collected from children at the age of 4 years and analyzed for the following compounds. A list of the chemicals studied is shown in Table 1.

#### 2.2.1. Organochlorine Pesticides and Polychlorinated Biphenyls

Non-fasting < 10 mL blood samples were collected in silicone gel separator vacutainers (Becton Dickinson, Cowley, Oxford, UK) and centrifuged within 2 h at 2500 rpm for 10 min. Then, serum samples were aliquoted and stored at −80 °C until assayed. The analysis was conducted in the Mass Spectrometry and Dioxin Analysis Laboratory (INRASTES, NCSR “Demokritos”) by the isotope dilution method using ^13^C labeled analogs for all compounds. The quantification of the examined compounds (HCB, DDT, DDE, and PCB 105, 114, 118, 123, 156, 157, 167, 189, 28, 52, 101, 138, 153, 180) was performed with High-Resolution Gas Chromatography-High-Resolution Mass Spectrometry (Electron-Impact) in Multiple Ion Detection mode, using previously described methods [42]. The concentrations were expressed in pg/mL serum. Concentrations below the limit of detection (LOD; 0.2 pg/mL for all monitored analytes) were substituted by the value of LOD divided by the square root of 2. OCPs were at detectable levels in every sample, while for PCBs detectability ranged from 55.3% to 100% of the samples. We calculated the total sum of all analyzed PCB congeners as well as the sum of indicator PCBs (28, 52, 101, 138, 153, 180). We adjusted for serum lipid concentrations (cholesterol and triglycerides) as continuous variables in all multivariable models to reduce potential biases from automatic lipid adjustment [43]. Analyses of serum lipid concentrations were conducted using standard enzymatic methods (Medicon Hellas S.A., Athens, Greece) on an automatic Olympus AU5400 high-volume chemistry analyzer (America, Inc., Melville, NY, USA). All chemicals were treated as continuous variables on a log_2_ scale, to account for right-skewness.

#### 2.2.2. Phthalates and Organophosphate Pesticides

Urine samples from children of 4 years of age were analyzed at the Department of Chemistry of the University of Crete. Total levels of seven phthalate metabolites (mEP, mnBP, miBP, mBzP, mEHP, mEHHP, and mEOHP) and six dialkyl phosphate metabolites (DAPs: DEP, DETP, DEDTP, DMP, DMTP, DMDTP) were monitored using previously described analytical protocols [40,44]. The limits of detection (LOD) for the examined phthalate metabolites ranged from 0.01 to 0.84, and for the organophosphate metabolites ranged from 0.02 to 0.24. Measurements below LOD were replaced by the LOD divided by the square root of 2. The percentage of samples with levels above the LOD were high for all phthalate metabolites, ranging from 97.9% to 100%. Regarding organophosphate metabolites, the percentage of samples with concentration levels above the LOD was high for DEP, DETP, and DMTP (91.5–98.9%), but low for DEDTP, DMP, and DMDTP (20.6–46.6%). We calculated the molar sum of DEHP metabolites (mEHP, mEHHP, mEOHP), High Molecular Weight phthalate (mEHP, mEHHP, mEOHP, mBzP), Low Molecular Weight phthalate metabolites (mEP, mnBP, miBP), as well as diethyl phosphate metabolites (DEs: DEP, DETP, DEDTP) and dimethyl phosphate metabolites (DMs: DMP, DMTP, DMDTP) by dividing metabolite concentrations with their molecular weight and summing across. Total DAPs represent the sum of diethyl and dimethyl metabolites and are used as a biomarker of total exposure to OPs [45]. In order to account for urine dilution, metabolite concentrations were divided by urinary creatinine levels, which were determined using the OLYMPUS 2700 immunoassay system (Beckman Coulter, Brea, CA, USA). All creatinine-adjusted metabolite concentrations were log_2_ transformed, due to right-skewed distribution.

### 2.3. Outcome Assessment

We evaluated children’s internalizing, externalizing, and ADHD symptoms at ages 4, 6, 11, and 15 years using maternal reports.

#### 2.3.1. Internalizing and Externalizing Symptoms

At age 4, mothers completed the Strengths and Difficulties Questionnaire (SDQ) [46], a concise tool that assesses children’s adaptive and maladaptive attributes. The SDQ consists of 25 items rated on a 3-point Likert scale. For the present analyses, two broad scales were used: (a) Internalizing difficulties, which encompasses emotional and peer problems and (b) Externalizing difficulties, which captures conduct problems and hyperactivity. The SDQ has been adapted to the Greek population [47]. In the subsequent follow-ups (ages 6, 11, 15) mothers completed the Child Behavior Checklist-Parent Report Form (CBCL) [48], a widely used instrument composed of 113 items rated on a 3-point Likert scale. For the current study, we utilized two broad-band scales: (a) Internalizing problems, which includes Anxiety/depression, Withdrawal/depression, and Somatic complaints syndrome scales and (b) Externalizing problems, which consists of Rule-breaking behavior and Aggressive behavior syndrome scales. The adaptation of the CBCL for the Greek population has been conducted by Roussos et al. [49]. In all used scales, higher scores reflect more difficulties.

#### 2.3.2. ADHD Symptoms

At age 4, the Attention Deficit Hyperactivity Disorder Test (ADHDT) [50] was filled out by mothers. It is a 36-item psychometric tool designed to evaluate ADHD-related symptoms. The sum of all items represents a total ADHD difficulties index, which was utilized in the present analyses. The ADHDT has been translated and adapted to the Greek population [51]. In subsequent assessments (ages 6, 11, 15 years) mothers reported their children’s ADHD symptoms using the Conners’ Parent Rating Scale-Revised: Short Form (CPRS-R: S) [52]. It is a short version consisting of 27 items rated on 4-point Likert scale. An ADHD index can be derived, with possible scores ranging from 0 to 36 and this index was employed in the current study. The CPRS-R: S was translated and cross-culturally adapted by the Rhea cohort team, according to the established methodology of forward and back-translation, along with pre-testing [53]. For both indices, higher scores indicate greater and more severe ADHD symptoms.

### 2.4. Statistical Analysis

Descriptive statistics were used to summarize the sample characteristics, the exposures and the outcomes of the study population. Means with standard deviations (SD) and frequencies with percentages were calculated for continuous and categorical variables, respectively. Pearson correlation coefficients were calculated to examine the correlation among chemicals. Sex differences in the distributions of exposures and outcomes were evaluated with the use of Student’s *t*-test or Mann–Whitney U. The differences between participants and non-participants were assessed via chi-square and t-tests or Mann–Whitney U test. To account for missing data, scale scores were prorated if fewer than 25% of items were missing. Because different instruments were used for outcome assessment at age 4 compared to later timepoints, we converted prorated scale scores to z-scores (Mean = 0, SD = 1) at each timepoint to harmonize the scales. The z-scores were used in all subsequent analyses.

The associations between each chemical exposure and internalizing, externalizing and ADHD symptom z-scores from ages 4 to 15 years were examined using Generalized Estimating Equation (GEE) models with an unstructured correlation matrix. Effect estimates are reported as beta coefficients with 95% confidence intervals (CIs). Various maternal and child characteristics, known or suspected to be linked to environmental exposures and children’s internalizing, externalizing, or ADHD symptoms, were considered potential confounding variables. Our models were adjusted for child sex (male, female), exact child age at outcome assessment (years), maternal age at childbirth (years), maternal education (low: up to 9 years of compulsory schooling, medium: post-secondary education, high: attained a university or technical/college degree), parity (nulliparous, multiparous), log-equivalized disposable household income in tertiles [54], child Body Mass Index (BMI; kg/m^2^) at 4 years, passive smoking at 4 years (never, ever), breastfeeding duration (months) and place of residence (urban, rural). Models for OCPs and PCBs were additionally adjusted for child serum lipids (cholesterol and triglycerides). To evaluate effect modification by child sex, we introduced in each model a multiplicative interaction term between each exposure and sex and then, using these models, we calculated the respective effect estimates for each sex.

Secondary analyses examined potential age-varying effects by including an interaction term between each exposure variable and the child’s age at outcome assessment (4, 6, 11 and 15 years) in the models. Effect estimates for each timepoint and the corresponding *p*-value for the age interaction were obtained from these models. In a subset of participants, maternal levels of chemicals were available. To evaluate the independent role of postnatal exposure, we adjusted GEE models for maternal levels of chemicals. Specifically, models for child exposure to OCPs and PCBs were adjusted for maternal levels of HCB, DDE, PCBs 118, 138, 153, 156, 170 and 180, and models for child phthalate exposure were adjusted for maternal levels of phthalates (HMW and LMW). Finally, we conducted sensitivity analyses: (a) Excluding children with a diagnosis of learning disability or ADHD (N = 29), and (b) Excluding preterm born children (<37 gestational weeks, N = 48).

The significance level was set at *p* < 0.05, and for all tests a two-sided alternative hypothesis was used. Statistical analyses were performed with Stata software, version 16.0 (Stata Corp, College Station, TX, USA).

## 3. Results

### 3.1. Descriptive Characteristics of the Study Population

The sample characteristics (N = 387) are presented in Table 2. Participating mothers were on average 30.2 years old (±4.7) at the time of childbirth, and the vast majority were Greek (96.1%). Approximately half of the mothers had acquired medium educational level (52.2%), while 38.0% had high level of education. A slightly higher percentage of mothers were multiparous (54.0%) compared to nulliparous (46.0%) mothers. Regarding child characteristics, 55.0% of the children were male and 45.0% were female. The average birthweight was 3204.3 g (±443.9), and the mean gestational age was 38.2 weeks (±1.6). Forty-eight children (12.4%) were born preterm. The median breastfeeding duration was 3 months. Most children (72.9%) lived in urban areas at the age of 4 and 43.4% were exposed to passive smoking. By the age of 15, twenty children (5.2%) had been diagnosed with learning disabilities and nine children (2.3%) with ADHD. No sex differences were observed in socio-demographic characteristics, except for birthweight which was higher among boys (*p* < 0.001).

Mothers in our sample were more likely to have higher education and household income than non-participating mothers. Children who participated had slightly higher birthweights, were breastfed for longer periods, and were more likely to have attended nursery before age 2. Regarding exposures, children in our sample were exposed to higher levels of DDE and HMW phthalates than non-participants (Table S1).

The concentrations of the examined biomarkers are shown in Table 3. For each chemical class, the highest concentrations were observed for DDE among OCPs, PCB153 among PCBs, mEP among phthalate metabolites, and DMTP among OPs. No significant sex differences were detected in exposure levels, except for miBP, which was found at higher concentrations in females (*p* = 0.022). Correlations among biomarker concentrations are presented in heatmap form in Figure S1. In brief, significant positive correlations were observed within the same chemical groups, as well as among phthalates and OPs.

With respect to outcome distribution (Table 4), girls exhibited significantly lower levels of emotional symptoms at age 4, but higher levels at age 15 compared to boys. As expected, boys demonstrated higher externalizing symptoms at ages 4, 6, and 11 years, as well as elevated ADHD symptoms across all follow-up assessments.

### 3.2. Childhood Exposure to Chemicals and Mental Health Symptoms from Childhood to Adolescence

The associations between chemical exposures at 4 years of age and internalizing, externalizing, and ADHD symptoms from early childhood to adolescence, estimated by GEE models, are presented in Table 5. No significant associations were observed when analyzing the overall sample. However, the sex-specific analysis revealed significant results. Specifically, within the class of organochlorines, exposure to HCB and DDE was associated with increased behavioral symptoms across ages in females [beta (95% CI): 0.20 (0.04, 0.37), *p*-interaction = 0.013 and 0.11 (0.01, 0.21), *p*-interaction = 0.001, respectively]. Moreover, a significant interaction with sex (*p* = 0.018) suggested that HCB exposure had opposite effects on ADHD symptoms in boys and girls. However, the effect estimates for each sex did not reach statistical significance. HCB exposure was also associated with decreased internalizing symptoms among boys [beta (95% CI): −0.13 (−0.25, −0.01)], though the interaction with sex was non-significant (*p* = 0.368). No significant associations were found for PCBs.

Among phthalate metabolites, exposure to LMW phthalates was associated with higher internalizing symptoms in girls [beta (95% CI): 0.15 (0.04, 0.26)], although the interaction with sex was not statistically significant (*p* = 0.054). Furthermore, LMW phthalate exposure was linked to increased externalizing and ADHD-related difficulties in girls [beta (95% CI): 0.13 (0.01, 0.25) and 0.13 (0.02, 0.24), respectively; *p*-interaction = 0.030 and 0.008]. For boys, the corresponding effect estimates were close to null for internalizing symptoms and negative, though not significant, for externalizing and ADHD symptoms.

Significant interactions with sex were also observed for OPs in relation to externalizing and ADHD symptoms. In girls, higher concentrations of diethyl (DE), dimethyl (DM), and dialkyl phosphate (DAP) metabolites were associated with elevated behavioral symptoms [beta (95% CI): 0.10 (0.02, 0.17), 0.08 (0.01, 0.16), and 0.13 (0.04, 0.22), respectively; *p*-interaction = 0.004, 0.019, and 0.003], and increased ADHD-related symptoms [beta (95% CI): 0.08 (0.01, 0.15), 0.09 (0.01, 0.16), and 0.12 (0.04, 0.20), respectively; *p*-interaction = 0.009, 0.014, and 0.003]. In boys, the corresponding effect estimates were in the opposite direction but did not reach statistical significance.

### 3.3. Secondary and Sensitivity Analyses

Furthermore, we investigated the age-specific associations between chemical exposures and child symptoms, identifying some time patterns (Table S2). Specifically, significant interactions of PCB exposure with age indicated that the exposure effect on ADHD symptoms was more pronounced at 6 years of age. Conversely, the association between OP exposure and internalizing symptoms was particularly evident at 15 years of age, although age interactions did not reach statistical significance.

In models further adjusted for prenatal exposure (Table S3), analysis of the overall sample showed that HCB exposure was linked to reduced internalizing symptoms [beta (95% CI): −0.10 (−0.19, −0.00)], while DEHP metabolites were related to elevated externalizing difficulties [beta (95% CI): 0.13 (0.01, 0.25)]. In models with sex interaction, no meaningful changes were observed for OCPs. For LMW phthalate exposure, effect estimates were slightly magnified, and significant inverse associations emerged for boys. However, these changes can be attributed to the significant reduction in sample size (approximately 75%) in this analysis.

Sensitivity analyses (Table S4) excluding children diagnosed with learning disabilities or ADHD and prematurely born children did not substantially change our results.

## 4. Discussion

In this longitudinal study, we explored the association of childhood exposure to a wide range of endocrine disrupting and neurotoxic chemicals with the developmental trajectories of internalizing, externalizing, and ADHD symptoms up to adolescence. Overall, our results revealed sex-specific associations, with the adverse effects of HCB, DDE, phthalates, and OPs on emotional and behavioral development being particularly observed in girls.

### 4.1. Organochlorine Pesticides

Our findings indicate that exposure to HCB and DDE is associated with increased behavioral symptoms among girls from early childhood through adolescence. A systematic investigation of OCPs revealed that children in the Rhea cohort were exposed to DDT/DDE in utero and through breastfeeding and diet during their early years, with exposure levels found to be higher compared to other countries [14]. Studies on childhood exposure to organochlorines are limited to those estimating exposure using pharmacokinetic models for the first two years of life [18,19,20] producing inconclusive results. In this context, sex differences are seldom explored, and the underlying mechanisms are not completely understood. However, animal studies have reported that DDE can act as antagonist on the androgen receptors [55]. In addition, endosulfan, an organochlorine insecticide similar to DDT, has been reported to decrease dopamine levels in the striatum of males and increase them in females [56]. Alterations in dopaminergic activity are implicated in ADHD [57], while disruption of brain monoamines by organochlorines may contribute to the observed sex-specific behavioral effects [58]. For example, one animal study reported that HCB exposure led to changes in norepinephrine, serotonin and dopamine concentrations, with effects varying by sex, dose, and specific brain region [59].

### 4.2. Phthalates

Moreover, higher concentrations of LMW phthalate metabolites in urine samples at age 4 years were associated with elevated internalizing, externalizing, and ADHD-related symptoms across development in girls. This finding is consistent with results from a longitudinal study in Cincinnati, USA, which identified more prominent associations between childhood exposure to mEP, a LMW phthalate, and internalizing and externalizing problems in girls [26]. Similarly, a cross-sectional Korean study reported that higher urine concentrations of mnBP were linked to increased thought and attention problems in females, although no sex differences were detected in total scores [29]. Furthermore, Daniel et al. [38] demonstrated that the adverse effects of phthalates differ between boys and girls, depending on the specific metabolite and behavioral aspect, providing further support to the sex-specific nature of these associations. The potential mechanisms through which phthalates exert their effects include the disruption of hormonal homeostasis, particularly of gonadal hormones, which are essential for brain function and sex-specific neurodevelopment [36]. Several phthalates have been shown to exhibit anti-androgenic properties [60] by interfering with aromatase activity, thereby affecting the sexual differentiation of the brain [61]. In addition, phthalates can interfere with thyroid function, with evidence suggesting that this effect is more pronounced in females [62,63]. This mechanism might also explain the sex differences in the behavioral effects of phthalates. However, further research is warranted to better understand the biological pathways underlying the effects of phthalates.

### 4.3. Organophosphate Pesticides

Finally, our analysis revealed another sex-specific association, with child exposure to OPs being linked to elevated externalizing and ADHD symptoms only among girls. Contaminated food has been identified as the primary source of OP exposure among children in the Rhea cohort [40]. Evidence on the behavioral effects of child pesticide exposure remains inconsistent. While some studies have demonstrated associations with ADHD-related behaviors [32,33] and emotional problems [34], others have found null or inverse associations [24,35]. However, it must be noted that direct comparisons with these studies cannot be made, due to differences in research design (e.g., cross-sectional studies), variations in the timing of exposure and outcome assessment, and in some cases the lack of biomarker-based exposure measurements. Evidence about sex differences is largely inconclusive. The effects of OPs are primarily attributed to acetylcholinesterase inhibition [64], which leads to the synaptic accumulation of acetylcholine, a neurotransmitter with significant role in cell proliferation, migration, and synapse formation [65,66]. Additional biological effects have also been proposed, including impaired glucose homeostasis, altered expression and function of transcription factors involved in cell differentiation and apoptosis, and epigenetic changes [67,68]. These mechanisms, along with the potential disruptions in sex and adrenal hormones caused by pesticides [69], may contribute to the varying effects observed between males and females.

### 4.4. Strengths and Limitations

This study has several strengths. First, it was conducted within the prospective mother-child Rhea cohort, allowing us to examine the effects of childhood exposure to a wide range of environmental chemicals on developmental trajectories across four timepoints, from early childhood to adolescence. Second, we used biomarkers for exposure assessment, providing a direct and reliable measure of exposure to all investigated chemicals, including organochlorine pesticides, polychlorinated biphenyls, phthalates, and organophosphate pesticides. Third, we focused on three composite scores for common childhood and adolescent difficulties—internalizing, externalizing, and ADHD symptoms—derived from valid and reliable psychometric instruments.

However, there are several limitations to consider. Although we did not observe major differences between participants included in our analysis and those excluded due to non-available data on both exposure and outcome, using a sub-sample of the initial population might have introduced selection bias in our analyses. Furthermore, the use of different instruments for outcome assessment may lead to inconsistencies in measurement, potentially affecting the accuracy of association estimates. However, evidence suggests that SDQ and CBCL are correlated and assess similar constructs [70,71], and data harmonization was performed using z-scores to minimize potential discrepancies. Another concern is that child symptoms were reported by mothers, which might have influenced the outcome assessment. Moreover, we acknowledge as a limitation of the study the lack of biomarkers of neurotoxicity or endocrine disruption (e.g., hormone levels, thyroid function, cytokines). Future studies integrating biological measurements of such indicators could offer valuable insights into the observed behavioral outcomes and help clarify the sex-specific effects identified in our analyses. In addition, further evidence on how environmental chemicals disrupt hormonal balance, especially estrogenic and androgenic pathways, can be critical for understanding their differential effects in boys and girls. Finally, although our models were adjusted for important confounding variables, the possibility of residual confounding remains.

## 5. Conclusions

Our study reveals that exposure to environmental chemicals at age 4 can have lasting adverse effects from early childhood through adolescence, particularly in girls. The robust sex-specific associations underscore the importance of considering sex as a key factor in environmental health research. Additionally, these findings highlight the need for further investigation into the biological mechanisms driving the differential effects of chemicals between males and females. Understanding these mechanisms is crucial for developing evidence-based policies and strategies aimed at minimizing children’s exposure to toxic chemicals, thereby protecting this vulnerable population.

## Figures and Tables

**Table 1:** List of studied chemicals and abbreviations.

Chemical	Abbreviation
**Organochlorine pesticides**	OCPs
Hexachlorobenzene	HCB
Dichlorodiphenyltrichloroethane	DDT
Dichlorodiphenyldichloroethylene	DDE
**Polychlorinated biphenyls** (105, 114, 118, 123, 156, 157, 167, 189)	PCBs
**Indicator Polychlorinated biphenyls** (28, 52, 101, 138, 153, 180)	ind-PCBs
**Phthalate metabolites**	
Mono-ethyl phthalate	mEP
Mono-n-butyl phthalate	mnBP
Mono-iso-butyl phthalate	miBP
Mono-benzyl phthalate	mBzP
Mono-2-ethyl-hexyl phthalate	mEHP
Mono-2-ethyl-5-hydroxy-hexyl phthalate	mEHHP
Mono-2-ethyl-5-oxo-hexyl phthalate	mEOHP
**Sums of phthalate metabolites**	
Di-2-ethyl-hexyl phthalate (mEHP, mEHHP, mEOHP)	DEHP
High Molecular Weight phthalate (mEHP, mEHHP, mEOHP, mBzP)	HMW
Low Molecular Weight phthalate (mEP, mnBP, miBP)	LMW
**Organophosphate metabolites**	OPs
Di-ethyl phosphate	DEP
Di-ethyl-thio phosphate	DETP
Di-ethyl-di-thio phosphate	DEDTP
Di-methyl phosphate	DMP
Di-methyl-thio phosphate	DMTP
Di-methyl-di-thio phosphate	DMDTP
**Sums of organophosphate metabolites**	
Di-ethyl phosphate metabolites (DEP, DETP, DEDTP)	DEs
Di-methyl phosphate metabolites (DMP, DMTP, DMDTP)	DMs
Dialkyl phosphate metabolites (DEP, DETP, DEDTP, DMP, DMTP, DMDTP)	DAPs

**Table 2:** Characteristics of the study population (N = 387).

	Overall (N = 387)	Male (N = 213)	Female (N = 174)	
	N (%) or Mean ±SD	N (%) or Mean ±SD	N (%) or Mean ± SD	*p*-Value
**Maternal characteristics**				
**Age at delivery** (years)	30.2 ± 4.7	30.0 ± 4.6	30.4 ± 4.8	0.398
**Ethnicity**				0.356
Non-Greek	15 (3.9)	10 (4.7)	5 (2.9)	
Greek	372 (96.1)	203 (95.3)	169 (97.1)	
**Education**				0.084
Low	38 (9.8)	18 (8.5)	20 (11.5)	
Medium	202 (52.2)	122 (57.3)	80 (46.0)	
High	147 (38.0)	73 (34.3)	74 (42.5)	
**Smoking** (12th gestational week)				0.759
No	321 (84.9)	176 (85.4)	145 (84.3)	
Yes	57 (15.1)	30 (14.6)	27 (15.7)	
**Parity**				0.843
Nulliparous	178 (46.0)	97 (45.5)	81 (46.6)	
Multiparous	209 (54.0)	116 (54.5)	93 (53.4)	
**Household income** (€) ^a^	1046.2 (829.8, 1324.4)	1033.7 (817.0, 1292.6)	1089.4 (849.2, 1356.5)	0.113
1st tertile (449–766 €)	64 (16.5)	37 (17.4)	27 (15.5)	0.227
2nd tertile (767–1078 €)	118 (30.5)	73 (34.3)	45 (25.9)	
3rd tertile (1079–2242 €)	154 (39.8)	77 (36.2)	77 (44.3)	
Unknown	51 (13.2)	26 (12.2)	25 (14.4)	
**Child characteristics**				
**Delivery Type**				0.127
Vaginal	199 (51.4)	117 (54.9)	82 (47.1)	
C-section	188 (48.6)	96 (45.1)	92 (52.9)	
**Birthweight** (g)	3204.3 ± 443.9	3288.9 ± 449.6	3101.1 ± 415.2	**<0.001**
**Gestational age** (weeks)	38.2 ± 1.6	38.3 ± 1.5	38.1 ± 1.6	0.270
**Preterm birth**				0.464
No	338 (87.6)	188 (88.7)	150 (86.2)	
Yes	48 (12.4)	24 (11.3)	24 (13.8)	
**Breastfeeding duration** (months) ^a^	3.0 (1.0, 6.4)	3.0 (1.0, 7.0)	3.0 (1.0, 6.0)	0.780
**Residence at 4 years**				0.856
Rural	105 (27.1)	57 (26.8)	48 (27.6)	
Urban	282 (72.9)	156 (73.2)	126 (72.4)	
**Passive smoking at 4 years**				0.254
No	219 (56.6)	115 (54.0)	104 (59.8)	
Yes	168 (43.4)	98 (46.0)	70 (40.2)	
**Nursery before 2 years**				0.950
No	293 (75.7)	161 (75.6)	132 (75.9)	
Yes	94 (24.3)	52 (24.4)	42 (24.1)	
**Nursery before 4 years**				0.738
No	49 (12.7)	28 (13.2)	21 (12.1)	
Yes	337 (87.3)	184 (86.8)	153 (87.9)	
**Diagnosis**				0.103
None	358 (92.5)	193 (90.6)	165 (94.8)	
Learning disabilities	20 (5.2)	12 (5.6)	8 (4.6)	
ADHD	9 (2.3)	8 (3.8)	1 (0.6)	
**Exact age at follow-up**				
4 years	4.2 ± 0.2	4.2 ± 0.2	4.2 ± 0.2	0.899
6 years	6.5 ± 0.3	6.5 ± 0.3	6.5 ± 0.2	0.666
11 years	11.0 ± 0.3	11.0 ± 0.4	11.0 ± 0.3	0.779
15 years	14.9 ± 0.4	14.9 ± 0.4	14.9 ± 0.4	0.627
**BMI at 4 years** (kg/m^2^)	16.4 ± 1.9	16.5 ± 1.9	16.4 ± 1.8	0.522

Abbreviation: BMI: Body Mass Index. ^a^ Presented data is median (25th–75th percentiles). Bold font indicates *p* < 0.05.

**Table 3:** Descriptive summary of biomarker concentrations measured in children according to chemical classes (N = 387).

Chemical	N	GM (95% CI)	Mean	SD	Min	Percentiles			Max	LOD	% > LOD
						25th	50th	75th			
**Organochlorine pesticides (pg/mL)**											
HCB	295	65.9 (61.0, 71.1)	92.9	137.3	16.0	44.0	58.0	84.0	1007.0	0.20	100
DDT	295	28.3 (25.5, 31.3)	43.5	57.5	1.0	17.0	27.0	49.0	516.0	0.20	100
DDE	295	676.3 (603.3, 758.1)	1173.6	1822.9	79.0	315.0	575.0	1400.0	22,603.0	0.20	100
**Polychlorinated biphenyls (pg/mL)**											
PCB105	295	1.7 (1.5, 1.9)	3.0	8.0	<0.2	1.1	1.6	2.4	92.7	0.20	99.3
PCB114	295	0.4 (0.4, 0.5)	1.1	3.5	<0.2	0.2	0.4	0.8	41.7	0.20	75.3
PCB118	295	6.4 (5.9, 7.0)	10.0	21.2	0.3	4.4	5.8	9.0	258.7	0.20	100
PCB123	295	0.6 (0.6, 0.7)	1.0	2.1	<0.2	0.4	0.6	0.9	24.3	0.20	94.9
PCB156	295	2.4 (2.2, 2.7)	3.7	4.8	<0.2	1.3	2.5	4.4	60.8	0.20	99.3
PCB157	295	1.1 (1.0, 1.3)	2.2	3.2	<0.2	0.5	1.0	2.6	33.4	0.20	92.9
PCB167	295	0.7 (0.6, 0.8)	1.1	1.6	<0.2	0.4	0.7	1.2	14.6	0.20	88.8
PCB189	295	0.3 (0.3, 0.3)	0.7	2.1	<0.2	0.1	0.2	0.4	23.9	0.20	55.3
PCB28	295	32.8 (30.3, 35.4)	62.1	211.1	9.4	23.3	29.6	37.1	2359.9	0.20	100
PCB52	295	3.6 (3.1, 4.2)	25.1	138.9	<0.2	1.7	2.7	4.9	1679.7	0.20	99.7
PCB101	295	4.0 (3.6, 4.5)	11.9	61.5	0.5	2.2	3.7	6.4	773.8	0.20	100
PCB138	295	19.6 (17.8, 21.5)	29.6	42.5	1.8	10.6	17.0	33.9	493.0	0.20	100
PCB153	295	38.0 (34.6, 41.7)	57.1	86.0	2.6	21.5	33.3	58.5	1146.6	0.20	100
PCB180	295	24.0 (21.9, 26.3)	34.8	41.1	3.6	13.5	21.2	40.9	333.6	0.20	100
**Sums of polychlorinated biphenyls (pg/mL)**											
ΣPCBs	295	156.7 (144.3, 170.1)	243.3	511.0	46.7	97.8	139.0	217.7	5758.8		
ind-PCBs	295	140.6 (129.2, 153.1)	224.1	479.7	40.0	90.0	120.0	200.0	5370.0		
**Phthalate metabolites (μg/g creatinine)**											
mEP	281	62.3 (55.1, 70.5)	128.6	281.8	6.2	14.9	26.8	49.2	695.0	0.40	100.0
mnBP	281	21.4 (18, 25.5)	44.8	74.2	<0.25	31.1	51.9	77.6	671.4	0.25	97.9
miBP	281	43.1 (37.9, 49)	62.8	57.4	<0.41	3.9	7.1	13.1	313.6	0.41	99.3
mBzP	281	7.6 (6.6, 8.7)	15.2	27.8	<0.02	6.1	11.0	20.1	300.7	0.02	99.6
mEHP	281	11.7 (10.5, 12.9)	19.0	30.7	2.0	28.5	40.5	68.9	2246.1	0.84	100.0
mEHHP	281	45.8 (41.6, 50.4)	77.1	189.5	2.7	23.2	35.9	59.6	1652.7	0.01	100.0
mEOHP	281	37.2 (33.6, 41.2)	62.4	141.9	1.8	23.5	35.8	59.7	1652.7	0.18	100.0
**Sums of phthalate metabolites (micromoles/g creatinine)**											
DEHP	281	0.3 (0.3, 0.4)	0.5	1.2	0.03	0.2	0.3	0.5	14.1		
HMW	281	0.4 (0.4, 0.4)	0.6	1.2	0.03	0.2	0.4	0.6	14.1		
LMW	281	0.8 (0.7, 0.9)	1.1	1.6	0.05	0.5	0.8	1.2	14.8		
**Organophosphate metabolites (μg/g creatinine)**											
DEP	281	3.2 (2.7, 3.9)	8.0	14.2	<0.04	1.7	3.5	7.8	145.5	0.04	94.3
DETP	281	1.4 (1.2, 1.7)	3.6	6.1	<0.03	0.6	1.8	4.5	72.6	0.03	91.5
DEDTP	281	0.05 (0.04, 0.06)	0.1	0.2	<0.03	0.0	0.0	0.1	1.4	0.03	20.6
DMP	281	0.4 (0.3, 0.4)	0.6	1.4	<0.24	0.2	0.3	0.5	21.3	0.24	24.6
DMTP	281	6.3 (5.4, 7.4)	18.3	66.0	<0.02	2.6	5.8	14.5	976.9	0.02	98.9
DMDTP	281	0.2 (0.2, 0.2)	1.6	7.3	<0.05	0.1	0.1	0.5	73.8	0.05	46.6
**Sums of organophosphate metabolites (nanomoles/g creatinine)**											
DEs	281	39.1 (34.5, 44.4)	73.3	121.2	0.7	19.4	36.3	77.9	1371.1		
DMs	281	55.0 (47.8, 63.1)	143.5	492.8	2.3	24.0	45.7	112.6	7158.2		
DAPs	281	108.8 (96.5, 122.8)	216.8	583.3	9.7	51.8	95.8	203.3	8529.3		

Abbreviations: GM: Geometric Mean; LOD: Limit of Detection. ΣPCBs: sum of all analyzed PCBs; ind-PCBS: sum of indicator PCBs (PCB 28, 52, 101, 138, 153, 180); DEHP: molar sum of mEHP, mEHHP, mEOHP; HMW: molar sum of mEHP, mEHHP, mEOHP, mBzP; LMW: molar sum of mEP, mnBP, miBP; DEs: molar sum of DEP, DETP, DEDTP; DMs: molar sum of DMP, DMTP, DMDTP; DAPs: sum of DEs and DMs.

**Table 4:** Outcome distribution (raw scores; N = 387).

	Overall		Males		Females		
	N	Mean (SD)	N	Mean (SD)	N	Mean (SD)	*p*-Value
**Internalizing symptoms**							
SDQ—4 years	376	3.1 (2.4)	204	3.4 (2.5)	172	2.8 (2.3)	**0.009**
CBCL—6 years	328	5.8 (4.1)	178	6.2 (4.3)	150	5.4 (3.9)	0.095
CBCL—11 years	265	6.9 (5.5)	145	7.2 (5.9)	120	6.5 (5.0)	0.291
CBCL—15 years	327	6.8 (5.6)	175	5.8 (5.0)	152	8.0 (6.1)	**0.001**
**Externalizing symptoms**							
SDQ—4 years	375	5.4 (3.2)	204	5.9 (3.4)	171	4.7 (2.8)	**<0.001**
CBCL—6 years	330	8.4 (6.1)	179	9.5 (6.3)	151	7.1 (5.7)	**<0.001**
CBCL—11 years	265	6.8 (5.8)	145	7.7 (6.5)	120	5.8 (4.7)	**0.006**
CBCL—15 years	326	6.4 (6.0)	175	6.5 (6.1)	151	6.3 (5.8)	0.744
**ADHD symptoms**							
ADHDT—4 years	374	14.7 (12.0)	203	16.6 (13.4)	171	12.4 (9.7)	**0.001**
CPRS-R: S—6 years	326	8.7 (5.4)	178	9.9 (5.7)	148	7.4 (4.7)	**<0.001**
CPRS-R: S—11 years	266	8.2 (5.5)	146	9.1 (5.9)	120	7.2 (4.7)	**0.004**
CPRS-R: S—15 years	327	7.9 (5.7)	175	8.8 (6.0)	152	6.9 (5.2)	**0.002**

Abbreviations: ADHDT: Attention Deficit Hyperactivity Disorder Test; CBCL: Child Behavior Checklist; CPRS-R: S: Conners’ Parent Rating Scale-Revised: Short Form; SDQ: Strengths and Difficulties Questionnaire. Bold font indicates *p* < 0.05.

**Table 5:** Associations of child exposure to environmental chemicals with internalizing, externalizing, and ADHD symptoms across 4 to 15 years.

	Overall ^a^			Males ^b^		Females ^b^		*p*-Interaction with
Exposure	N	Beta (95% CI)	*p*-Value	Beta (95% CI)	*p*-Value	Beta (95% CI)	*p*-Value	Sex ^b^
**Internalizing symptoms z-score**								
HCB	293	−0.10 (−0.19, 0.00)	0.054	**−0.13 (−0.25, −0.01)**	**0.036**	−0.05 (−0.19, 0.10)	0.540	0.368
DDT	293	−0.02 (−0.08, 0.04)	0.507	−0.05 (−0.13, 0.03)	0.191	0.03 (−0.07, 0.14)	0.523	0.197
DDE	293	0.03 (−0.04, 0.10)	0.394	−0.01 (−0.09, 0.08)	0.898	0.08 (−0.02, 0.18)	0.103	0.134
ΣPCBs	293	−0.03 (−0.13, 0.06)	0.491	−0.01 (−0.13, 0.11)	0.843	−0.06 (−0.18, 0.07)	0.384	0.593
ind-PCBs	293	−0.01 (−0.11, 0.08)	0.814	0.01 (−0.12, 0.13)	0.917	−0.03 (−0.15, 0.09)	0.633	0.644
DEHP	281	0.04 (−0.04, 0.11)	0.327	0.04 (−0.06, 0.14)	0.409	0.03 (−0.08, 0.14)	0.591	0.880
HMW	281	0.05 (−0.03, 0.12)	0.211	0.06 (−0.04, 0.16)	0.262	0.04 (−0.08, 0.15)	0.541	0.775
LMW	281	0.06 (−0.01, 0.14)	0.091	0.00 (−0.09, 0.10)	0.925	**0.15 (0.04, 0.26)**	**0.010**	0.054
DEs	281	−0.00 (−0.05, 0.05)	0.945	−0.03 (−0.11, 0.04)	0.365	0.03 (−0.04, 0.10)	0.416	0.224
DMs	281	0.02 (−0.03, 0.07)	0.439	0.01 (−0.05, 0.08)	0.674	0.03 (−0.05, 0.10)	0.477	0.785
DAPs	281	0.02 (−0.04, 0.07)	0.504	−0.00 (−0.08, 0.07)	0.902	0.05 (−0.03, 0.13)	0.244	0.335
**Externalizing symptoms z-score**								
HCB	292	0.04 (−0.07, 0.14)	0.480	−0.05 (−0.17, 0.08)	0.465	**0.20 (0.04, 0.37)**	**0.017**	**0.013**
DDT	292	0.01 (−0.06, 0.08)	0.777	−0.02 (−0.10, 0.07)	0.710	0.06 (−0.06, 0.17)	0.330	0.317
DDE	292	0.00 (−0.08, 0.08)	0.984	−0.08 (−0.18, 0.01)	0.065	**0.11 (0.01, 0.21)**	**0.025**	**0.001**
ΣPCBs	292	−0.00 (−0.10, 0.10)	0.969	−0.03 (−0.16, 0.10)	0.661	0.03 (−0.11, 0.16)	0.690	0.517
ind-PCBs	292	0.01 (−0.08, 0.11)	0.776	−0.02 (−0.15, 0.10)	0.718	0.05 (−0.08, 0.18)	0.413	0.350
DEHP	281	0.05 (−0.03, 0.13)	0.212	0.03 (−0.08, 0.13)	0.599	0.08 (−0.04, 0.20)	0.203	0.549
HMW	281	0.04 (−0.04, 0.12)	0.304	0.02 (−0.09, 0.12)	0.768	0.07 (−0.05, 0.19)	0.226	0.478
LMW	281	0.02 (−0.05, 0.10)	0.547	−0.05 (−0.14, 0.05)	0.370	**0.13 (0.01, 0.25)**	**0.039**	**0.030**
DEs	281	0.01 (−0.04, 0.07)	0.604	−0.06 (−0.14, 0.01)	0.104	**0.10 (0.02, 0.17)**	**0.017**	**0.004**
DMs	281	0.01 (−0.04, 0.06)	0.630	−0.04 (−0.10, 0.03)	0.259	**0.08 (0.01, 0.16)**	**0.036**	**0.019**
DAPs	281	0.03 (−0.03, 0.09)	0.359	−0.05 (−0.13, 0.03)	0.209	**0.13 (0.04, 0.22)**	**0.004**	**0.003**
**ADHD symptoms z-score**								
HCB	294	−0.02 (−0.12, 0.08)	0.674	−0.10 (−0.22, 0.02)	0.094	0.11 (−0.04, 0.27)	0.137	**0.018**
DDT	294	0.01 (−0.06, 0.07)	0.803	0.01 (−0.08, 0.09)	0.903	0.01 (−0.09, 0.12)	0.801	0.897
DDE	294	−0.02 (−0.10, 0.05)	0.584	−0.07 (−0.15, 0.02)	0.154	0.04 (−0.06, 0.14)	0.464	0.083
ΣPCBs	294	0.06 (−0.03, 0.16)	0.190	0.09 (−0.03, 0.21)	0.154	0.04 (−0.09, 0.16)	0.574	0.521
ind-PCBs	294	0.06 (−0.03, 0.16)	0.200	0.08 (−0.05, 0.20)	0.217	0.05 (−0.08, 0.17)	0.457	0.710
DEHP	280	0.06 (−0.01, 0.13)	0.120	0.08 (−0.02, 0.17)	0.135	0.04 (−0.07, 0.15)	0.518	0.607
HMW	280	0.05 (−0.03, 0.12)	0.226	0.06 (−0.04, 0.16)	0.238	0.03 (−0.08, 0.14)	0.630	0.675
LMW	280	0.01 (−0.06, 0.08)	0.761	−0.07 (−0.16, 0.02)	0.151	**0.13 (0.02, 0.24)**	**0.025**	**0.008**
DEs	280	0.01 (−0.04, 0.06)	0.664	−0.06 (−0.13, 0.02)	0.127	**0.08 (0.01, 0.15)**	**0.031**	**0.009**
DMs	280	0.02 (−0.03, 0.06)	0.485	−0.03 (−0.09, 0.03)	0.306	**0.09 (0.01, 0.16)**	**0.021**	**0.014**
DAPs	280	0.03 (−0.03, 0.08)	0.366	−0.05 (−0.12, 0.03)	0.207	**0.12 (0.04, 0.20)**	**0.004**	**0.003**

Internalizing, externalizing and ADHD symptoms are expressed as z-scores. All exposures are log_2_ transformed. Values were derived from generalized estimating equations (GEE) analyses with child measures at 4, 6, 11 and 15 years. ^a^ Models were adjusted for child sex, exact age at assessment, child lipids (cholesterol and triglycerides, only for models with OCPs and PCBs), maternal age at delivery, maternal education, parity, household income, child BMI at 4 years, passive smoking at 4 years, breastfeeding duration and place of residence. ^b^ Models also included a multiplicative interaction term between exposure and child sex (male, female). The *p*-interaction values correspond to the exposure-sex interaction term. Bold font indicates *p* < 0.05.

## Data Availability

Data supporting these findings are available upon request from the corresponding author K.K.

## Supplementary Materials

The following supporting information can be downloaded at: https://www.mdpi.com/article/10.3390/toxics13100854/s1, Table S1: Parental and offspring characteristics of the sample included in analyses (N = 387) compared to those excluded from analyses (N = 610); Figure S1: Pearsons’ correlation coefficients of biomarker concentrations (log_2_) measured in children (Ν = 387) within and between chemical groups; Table S2: Associations of child exposure to environmental chemicals with internalizing, externalizing, and ADHD symptoms at ages 4, 6, 11 and 15 years; Table S3: Associations of child exposure to environmental chemicals with internalizing, externalizing, and ADHD symptoms across 4 to 15 years, adjusted for prenatal exposure; Table S4: Associations of child exposure to environmental chemicals with internalizing, externalizing, and ADHD symptoms across 4 to 15 years, sensitivity analyses.

## Abbreviations

The following abbreviations are used in this manuscript:

ADHD	Attention-Deficit/Hyperactivity Disorder
ADHDT	Attention-Deficit/Hyperactivity Disorder Test
ASD	Autism Spectrum Disorder
BMI	Body Mass Index
CBCL	Child Behavior Checklist
CPRS-R:S	Conners’ Parent Rating Scale-Revised: Short Form
GEE	Generalized Estimating Equation
Ind-PCBs	Indicator Polychlorinated Biphenyls
LOD	Limit of Detection
OCPs	Organochlorine Pesticides
OPs	Organophosphate Pesticides
PCBs	Polychlorinated Biphenyls
SDQ	Strengths and Difficulties Questionnaire
