# Supplementary material for: Early Childhood Exposure to Endocrine Disrupting and Neurotoxic Chemicals: Associations with Internalizing and Externalizing Difficulties from Childhood to Adolescence in the Rhea Cohort, Crete, Greece
Source: Toxics. 2025 Oct 10;13(10):854. doi: 10.3390/toxics13100854 (PMC12568248; doi:10.3390/toxics13100854)
Supplement: Supplementary file 1 [file toxics-13-00854-s001.zip › toxics-3861572-supplementary.pdf]

Table S1. Parental and offspring characteristics of the sample included in analyses (N=387) compared to those excluded from analyses (N=610).

	Participants		Non-participants		
	**N**	**% or Mean ±SD**	**N**	**% or Mean ±SD**	**p-value**
Maternal characteristics					
**Age at delivery** (years)	387	30.2 ±4.7	602	29.8 (5.2)	0.327
**Ethnicity**					
Non-Greek	15	3.9	37	6.1	0.121
Greek	372	96.1	567	93.9	
**Education**					
Low	38	9.8	113	19.7	
Medium	202	52.2	293	51.0	<0.001
High	147	38.0	168	29.3	
**Smoking** (12th week)					
No	321	84.9	433	80.8	0.105
Yes	57	15.1	103	19.2	
**Parity**					
Nulliparous	178	46.0	271	47.1	0.729
Multiparous	209	54.0	304	52.9	
**Household income** [€, Median (25-75 pct)]	336	1046.2 (829.8, 1324.4)	438	936.9 (703.4, 1226.0)	<0.001
1st tertile (380-766€)	64	16.5	141	23.1	<0.001
2nd tertile (767-1078€)	118	30.5	143	23.4	
3rd tertile (1079-2242€)	154	39.8	154	25.2	
Unknown	51	13.2	172	28.2	
Child characteristics					
**Sex**					
Male	213	55.0	311	51.0	0.211
Female	174	45.0	299	49.0	
**Delivery Type**					
Vaginal	199	51.4	268	45.9	0.091
C-section	188	48.6	316	54.1	
**Birthweight** (kg)	384	3.2 ±0.4	572	3.1 ±0.5	0.003
**Gestational age** (weeks)	386	38.2 ±1.6	577	38.0 ±1.8	0.117
**Preterm birth**					0.098
No	338	87.6	483	83.7	
Yes	48	12.4	94	16.3	
**Breastfeeding duration** [months, Median (25-75 pct)]	387	3.0 (1.0, 6.4)	547	2.0 (0.5, 5.0)	<0.001
**Residence at 4 years**					
Rural	105	27.1	170	29.0	0.535
Urban	285	72.9	417	71.0	
**Passive smoking at 4 years**					
No	219	56.6	247	51.9	0.168
Yes	168	43.4	229	48.1	
**Nursery before 2 years**					
No	293	75.7	477	82.2	0.014
Yes	94	24.3	102	17.8	
**Nursery before 4 years**					
No	49	12.7	80	16.6	0.111
Yes	337	87.3	403	83.4	
**Diagnosis**					0.315
None	358	92.5	415	94.7	
Learning disabilities	20	5.2	18	4.1	
ADHD	9	2.3	5	1.1	
**BMI at 4 years** (kg/m^2)	387	16.4 ±1.9	455	16.5 ±2.0	0.565
Exposures		**Median (25-75 pct)**		**Median (25-75 pct)**	

HCB (pg/mL)	295	58.0 (44.0, 84.0)	72	49.5 (41.0, 86.5)	0.155
DDT (pg/mL)	295	27.0 (17.0, 49.0)	72	22.5 (15.0, 38.0)	0.076
DDE (pg/mL)	295	575.0 (315.0, 1400.0)	72	390.0 (257.5, 924.5)	0.008
ΣPCBs (pg/ml)	295	139.0 (97.8, 217.7)	72	112.8 (88.0, 197.9)	0.117
ind-PCBs (pg/mL)	295	120.0 (90.0, 200.0)	72	100.0 (75.0, 180.0)	0.116
DEHP (µmoles/g creatinine)	281	0.3 (0.2, 0.5)	204	0.3 (0.2, 0.5)	0.073
HMW (µmoles/g creatinine)	281	0.4 (0.2, 0.6)	204	0.3 (0.2, 0.5)	0.042
LMW (µmoles/g creatinine)	281	0.8 (0.5, 1.2)	204	0.7 (0.5, 1.3)	0.981
DEs (nmol/g creatinine)	281	36.3 (19.4, 77.9)	204	35.1 (16.8, 79.3)	0.677
DMs (nmol/g creatinine)	281	45.7 (24.0, 112.6)	204	48.6 (25.0, 109.3)	0.632
DAPs (nmol/g creatinine)	281	95.8 (51.8, 203.3)	204	99.3 (51.2, 204.9)	0.827

Abbreviations: BMI: Body Mass Index; pct: percentile.

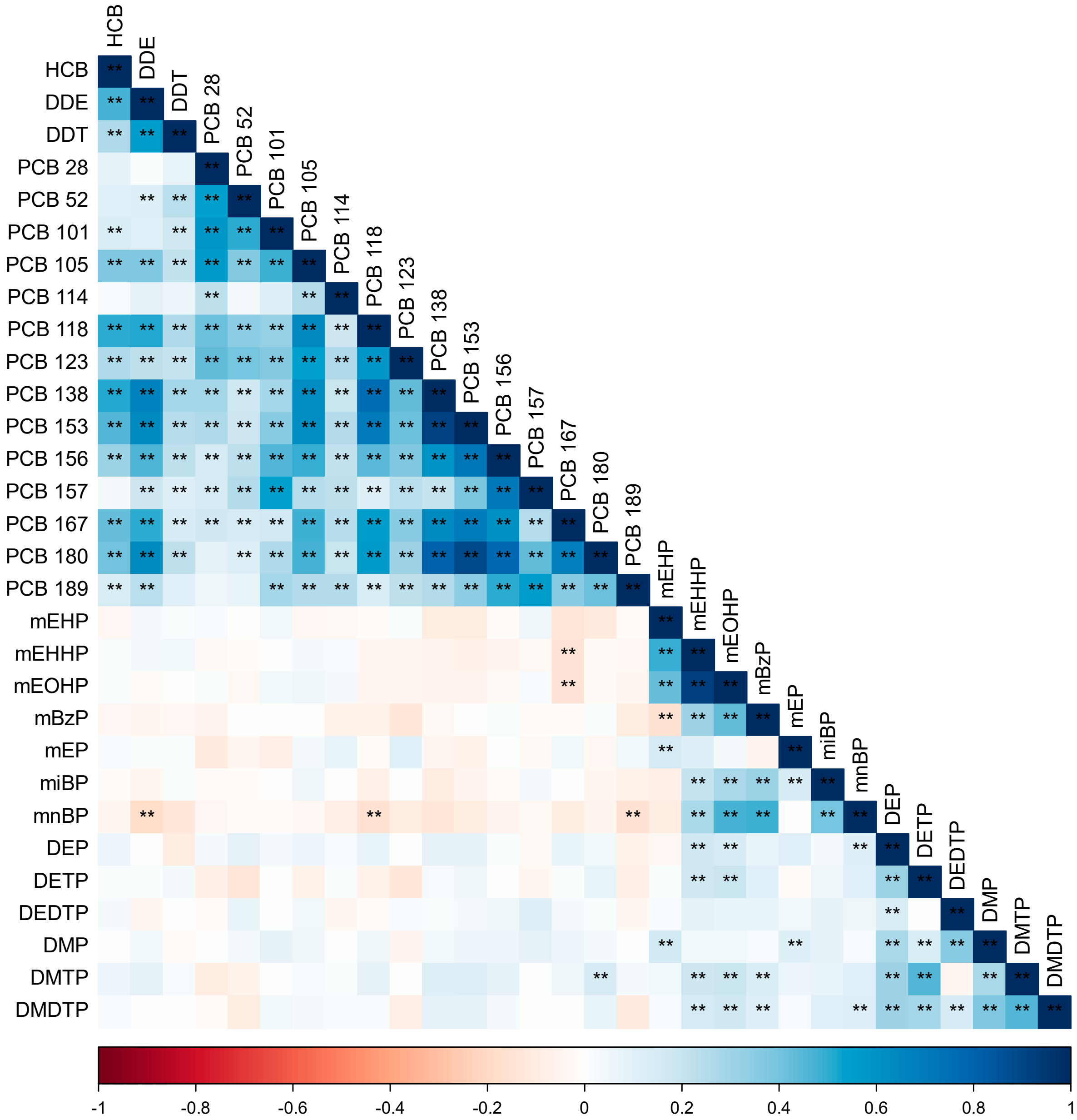

Figure S1. Pearsons' correlation coefficients of biomarker concentrations (log_2) measured in children (n=387) within and between chemical groups. The color indicates the strength of the correlation. The color scale is shown at the bottom.
* p < 0.05, ** p < 0.01.

Table S2. Associations of child exposure to environmental chemicals with internalizing, externalizing, and ADHD symptoms at ages 4, 6, 11 and 15 years.

		4 years		6 years		11 years		15 years		p-interaction
**Exposure**	**N**	**beta (95% CI)**	**p-value**	**beta (95% CI)**	**p-value**	**beta (95% CI)**	**p-value**	**beta (95% CI)**	**p-value**	**with age**
**Internalizing symptoms (z-score)**										
HCB	293	**-0.15 (-0.27, -0.02)**	**0.024**	-0.06 (-0.19, 0.07)	0.384	-0.11 (-0.24, 0.03)	0.121	-0.07 (-0.20, 0.06)	0.291	0.581
DDT	293	0.01 (-0.08, 0.10)	0.841	-0.02 (-0.11, 0.07)	0.656	-0.01 (-0.11, 0.08)	0.811	-0.07 (-0.16, 0.02)	0.150	0.534
DDE	293	0.01 (-0.08, 0.10)	0.772	0.03 (-0.06, 0.12)	0.523	0.02 (-0.08, 0.12)	0.707	0.07 (-0.03, 0.16)	0.174	0.716
ΣPCBs	293	-0.07 (-0.19, 0.05)	0.226	-0.05 (-0.17, 0.07)	0.434	-0.01 (-0.15, 0.12)	0.837	0.01 (-0.11, 0.14)	0.822	0.552
ind-PCBs	293	-0.04 (-0.16, 0.08)	0.485	-0.03 (-0.15, 0.09)	0.583	-0.00 (-0.14, 0.13)	0.981	0.04 (-0.08, 0.16)	0.535	0.569
DEHP	281	0.04 (-0.07, 0.15)	0.462	0.05 (-0.06, 0.17)	0.352	-0.05 (-0.17, 0.08)	0.474	0.08 (-0.03, 0.19)	0.159	0.395
HMW	281	0.02 (-0.08, 0.13)	0.647	0.10 (-0.01, 0.21)	0.078	-0.05 (-0.17, 0.07)	0.432	0.10 (-0.02, 0.21)	0.094	0.143
LMW	281	0.06 (-0.04, 0.16)	0.241	0.09 (-0.02, 0.20)	0.092	0.04 (-0.08, 0.15)	0.510	0.06 (-0.06, 0.17)	0.324	0.875
DEs	281	-0.06 (-0.13, 0.01)	0.108	0.01 (-0.07, 0.09)	0.779	-0.01 (-0.09, 0.08)	0.892	0.06 (-0.02, 0.13)	0.159	0.088
DMs	281	-0.01 (-0.08, 0.06)	0.823	0.02 (-0.05, 0.09)	0.597	-0.02 (-0.10, 0.06)	0.554	**0.08 (0.01, 0.15)**	**0.031**	0.114
DAPs	281	-0.04 (-0.12, 0.04)	0.345	0.02 (-0.06, 0.11)	0.555	-0.01 (-0.10, 0.08)	0.909	**0.09 (0.01, 0.17)**	**0.024**	0.050
**Externalizing symptoms (z-score)**										
HCB	292	0.01 (-0.12, 0.14)	0.861	0.11 (-0.02, 0.23)	0.100	-0.00 (-0.14, 0.13)	0.962	0.02 (-0.11, 0.16)	0.713	0.284
DDT	292	0.06 (-0.02, 0.15)	0.138	0.01 (-0.08, 0.09)	0.881	0.01 (-0.08, 0.10)	0.799	-0.05 (-0.14, 0.04)	0.247	0.089
DDE	292	-0.00 (-0.09, 0.09)	0.999	0.01 (-0.08, 0.10)	0.874	0.00 (-0.09, 0.10)	0.943	-0.01 (-0.10, 0.08)	0.862	0.985
ΣPCBs	292	-0.02 (-0.14, 0.09)	0.710	0.01 (-0.11, 0.13)	0.854	-0.04 (-0.17, 0.09)	0.547	0.03 (-0.09, 0.15)	0.594	0.625
ind-PCBs	292	0.00 (-0.12, 0.12)	0.995	0.02 (-0.09, 0.14)	0.685	-0.03 (-0.16, 0.10)	0.612	0.05 (-0.07, 0.17)	0.392	0.529
DEHP	281	0.08 (-0.03, 0.18)	0.159	0.07 (-0.04, 0.18)	0.194	0.01 (-0.11, 0.13)	0.889	0.03 (-0.08, 0.14)	0.593	0.679
HMW	281	0.04 (-0.06, 0.15)	0.416	0.08 (-0.03, 0.19)	0.151	0.01 (-0.12, 0.13)	0.929	0.03 (-0.08, 0.14)	0.616	0.707
LMW	281	0.00 (-0.10, 0.11)	0.932	0.02 (-0.08, 0.13)	0.680	0.06 (-0.05, 0.17)	0.296	0.02 (-0.09, 0.13)	0.765	0.836
DEs	281	0.01 (-0.06, 0.09)	0.734	0.01 (-0.07, 0.08)	0.867	0.00 (-0.08, 0.08)	0.953	0.03 (-0.04, 0.11)	0.378	0.898
DMs	281	-0.01 (-0.08, 0.05)	0.723	0.03 (-0.04, 0.10)	0.451	-0.01 (-0.09, 0.07)	0.849	0.04 (-0.03, 0.11)	0.256	0.467
DAPs	281	0.01 (-0.07, 0.08)	0.869	0.03 (-0.05, 0.11)	0.491	0.03 (-0.06, 0.11)	0.575	0.05 (-0.03, 0.13)	0.197	0.773
**ADHD symptoms (z-score)**										
HCB	294	-0.04 (-0.17, 0.09)	0.528	0.04 (-0.09, 0.16)	0.566	-0.05 (-0.18, 0.08)	0.445	-0.04 (-0.17, 0.09)	0.574	0.508
DDT	294	0.07 (-0.01, 0.16)	0.101	0.01 (-0.07, 0.10)	0.815	0.02 (-0.07, 0.11)	0.724	-0.07 (-0.16, 0.02)	0.114	**0.031**
DDE	294	-0.04 (-0.13, 0.05)	0.364	-0.01 (-0.10, 0.07)	0.749	-0.02 (-0.12, 0.08)	0.695	-0.01 (-0.10, 0.09)	0.892	0.862
ΣPCBs	294	0.01 (-0.10, 0.13)	0.820	**0.17 (0.05, 0.28)**	**0.007**	0.03 (-0.10, 0.16)	0.629	0.04 (-0.08, 0.16)	0.498	**0.043**
ind-PCBs	294	0.01 (-0.11, 0.12)	0.882	**0.16 (0.04, 0.28)**	**0.009**	0.03 (-0.10, 0.16)	0.647	0.05 (-0.07, 0.17)	0.390	**0.047**
DEHP	280	0.09 (-0.02, 0.19)	0.094	0.05 (-0.06, 0.16)	0.382	0.06 (-0.06, 0.18)	0.306	0.03 (-0.08, 0.13)	0.653	0.808
HMW	280	0.07 (-0.04, 0.17)	0.212	0.04 (-0.07, 0.15)	0.453	0.05 (-0.07, 0.17)	0.431	0.02 (-0.09, 0.13)	0.721	0.918

LMW	280	0.02 (-0.08, 0.12)	0.705	-0.00 (-0.11, 0.10)	0.975	-0.01 (-0.12, 0.10)	0.832	0.04 (-0.07, 0.14)	0.520	0.900
DEs	280	-0.00 (-0.08, 0.07)	0.935	-0.03 (-0.10, 0.05)	0.518	0.02 (-0.06, 0.10)	0.639	0.06 (-0.02, 0.13)	0.153	0.352
DMs	280	-0.01 (-0.07, 0.06)	0.873	0.01 (-0.06, 0.08)	0.845	0.07 (-0.01, 0.14)	0.095	0.02 (-0.05, 0.09)	0.629	0.425
DAPs	280	-0.00 (-0.08, 0.08)	0.994	-0.00 (-0.08, 0.08)	0.932	0.08 (-0.01, 0.16)	0.091	0.04 (-0.04, 0.12)	0.284	0.342

Internalizing, externalizing and ADHD symptoms are expressed as z-scores. All exposures are $\log_2$ transformed.
Values were derived from generalized estimating equations (GEE) analyses with child measures at 4, 6, 11 and 15 years.
Models were adjusted for child sex, exact age at assessment, child lipids (cholesterol and triglycerides, only for models with OCPs and PCBs), maternal age at delivery, maternal education, parity, household income, child BMI at 4 years, passive smoking at 4 years, breastfeeding duration and place of residence. Models also included a multiplicative interaction term between exposure and child age at assessment (4, 6, 11, and 15 years). The p-interaction values correspond to the exposure-age interaction term.
Bold font indicates $p < 0.05$.

Table S3. Associations of child exposure to environmental chemicals with internalizing, externalizing, and ADHD symptoms across 4 to 15 years, **adjusted for prenatal exposure**.

	Overall [a]			Males [b]		Females [b]		p-interaction
**Exposure**	**N**	**beta (95% CI)**	**p-value**	**beta (95% CI)**	**p-value**	**beta (95% CI)**	**p-value**	**with sex [b]**
**Internalizing symptoms (z-score)**								
HCB	270	**-0.10 (-0.19, -0.00)**	**0.047**	**-0.12 (-0.24, -0.00)**	**0.044**	-0.06 (-0.20, 0.08)	0.412	0.491
DDT	270	-0.03 (-0.10, 0.03)	0.304	-0.07 (-0.15, 0.01)	0.096	0.03 (-0.08, 0.13)	0.618	0.161
DDE	270	0.03 (-0.06, 0.11)	0.516	-0.00 (-0.10, 0.09)	0.926	0.07 (-0.03, 0.18)	0.176	0.173
ΣPCBs	270	-0.01 (-0.10, 0.09)	0.900	0.02 (-0.10, 0.14)	0.729	-0.04 (-0.16, 0.09)	0.578	0.476
ind-PCBs	270	0.02 (-0.08, 0.11)	0.711	0.04 (-0.08, 0.17)	0.474	-0.01 (-0.13, 0.11)	0.895	0.494
DEHP	94	0.03 (-0.08, 0.13)	0.601	0.02 (-0.14, 0.18)	0.777	0.03 (-0.10, 0.17)	0.646	0.934
HMW	94	0.03 (-0.08, 0.13)	0.637	0.03 (-0.13, 0.19)	0.719	0.02 (-0.11, 0.16)	0.739	0.952
LMW	94	0.03 (-0.07, 0.13)	0.552	-0.09 (-0.23, 0.05)	0.191	**0.15 (0.01, 0.29)**	**0.030**	**0.013**
**Externalizing symptoms (z-score)**								
HCB	269	0.04 (-0.07, 0.14)	0.475	-0.05 (-0.17, 0.07)	0.432	**0.20 (0.04, 0.37)**	**0.016**	**0.011**
DDT	269	0.01 (-0.05, 0.08)	0.695	-0.02 (-0.10, 0.07)	0.667	0.07 (-0.04, 0.19)	0.211	0.205
DDE	269	0.01 (-0.08, 0.10)	0.775	-0.08 (-0.18, 0.02)	0.126	**0.14 (0.03, 0.26)**	**0.013**	**<0.001**
ΣPCBs	269	0.02 (-0.08, 0.12)	0.675	-0.01 (-0.14, 0.12)	0.862	0.06 (-0.08, 0.19)	0.402	0.423
ind-PCBs	269	0.04 (-0.06, 0.14)	0.436	-0.00 (-0.13, 0.13)	0.970	0.08 (-0.05, 0.21)	0.208	0.296
DEHP	94	**0.13 (0.01, 0.25)**	**0.034**	0.03 (-0.14, 0.21)	0.707	**0.20 (0.05, 0.35)**	**0.011**	0.161
HMW	94	0.12 (-0.01, 0.24)	0.063	0.01 (-0.17, 0.20)	0.884	**0.19 (0.04, 0.35)**	**0.016**	0.130
LMW	94	-0.05 (-0.17, 0.07)	0.389	**-0.29 (-0.44, -0.13)**	**<0.001**	**0.18 (0.03, 0.33)**	**0.019**	**<0.001**
**ADHD symptoms (z-score)**								
HCB	271	-0.02 (-0.12, 0.08)	0.734	-0.10 (-0.22, 0.02)	0.113	0.11 (-0.04, 0.26)	0.144	**0.023**
DDT	271	0.02 (-0.05, 0.09)	0.554	0.01 (-0.07, 0.10)	0.777	0.03 (-0.08, 0.15)	0.537	0.744
DDE	271	-0.01 (-0.10, 0.08)	0.821	-0.06 (-0.16, 0.04)	0.240	0.06 (-0.05, 0.17)	0.302	**0.047**
ΣPCBs	271	0.08 (-0.02, 0.18)	0.108	0.11 (-0.02, 0.23)	0.092	0.05 (-0.08, 0.18)	0.433	0.491
ind-PCBs	271	0.08 (-0.02, 0.18)	0.120	0.10 (-0.03, 0.23)	0.135	0.06 (-0.07, 0.19)	0.342	0.657
DEHP	93	0.09 (-0.04, 0.22)	0.168	0.06 (-0.13, 0.25)	0.549	0.11 (-0.05, 0.28)	0.182	0.673
HMW	93	0.08 (-0.06, 0.21)	0.257	0.03 (-0.16, 0.23)	0.731	0.11 (-0.06, 0.28)	0.216	0.572
LMW	93	-0.03 (-0.15, 0.10)	0.645	**-0.30 (-0.46, -0.15)**	**<0.001**	**0.24 (0.09, 0.40)**	**0.002**	**<0.001**

Internalizing, externalizing and ADHD symptoms are expressed as z-scores. All exposures are $\log_2$ transformed.
Values were derived from generalized estimating equations (GEE) analyses with child measures at 4, 6, 11 and 15 years.
[a] Models were adjusted for child sex, exact age at assessment, child lipids (cholesterol and triglycerides, only for models with OCPs and PCBs), maternal age at delivery, maternal education, parity, household income, child BMI at 4 years, passive smoking at 4 years, breastfeeding duration, place of residence, and the corresponding prenatal exposure levels. Specifically, models for child exposure to HCB, DDT, DDE, ΣPCBs and ind-PCBs were adjusted for maternal levels of HCB, DDE, PCBs 118, 138, 153, 156, 170 and 180. Models for child exposure to DEHP, HMW, and LMW were adjusted for maternal levels of phthalates (HMW and LMW).

[b] Models also included a multiplicative interaction term between exposure and child sex (male, female). The p-interaction values correspond to the exposure-sex interaction term.
Bold font indicates $p < 0.05$.

Table S4. Associations of child exposure to environmental chemicals with internalizing, externalizing, and ADHD symptoms across 4 to 15 years, **sensitivity analyses**.

	Exclude children with learning disabilities or ADHD diagnosis [a]					Exclude preterm born children [b]				
	Males		Females		p-interaction	Males		Females		p-interaction
Exposure	beta (95% CI)	p-value	beta (95% CI)	p-value	with sex	beta (95% CI)	p-value	beta (95% CI)	p-value	with sex
**Internalizing symptoms (z-score)**										
HCB	**-0.13 (-0.25, -0.01)**	**0.034**	-0.07 (-0.24, 0.10)	0.409	0.535	-0.12 (-0.25, 0.00)	0.058	-0.03 (-0.19, 0.12)	0.662	0.350
DDT	-0.05 (-0.13, 0.03)	0.245	0.03 (-0.08, 0.13)	0.638	0.283	-0.06 (-0.14, 0.03)	0.191	0.05 (-0.07, 0.16)	0.409	0.153
DDE	0.01 (-0.09, 0.10)	0.892	0.09 (-0.01, 0.19)	0.095	0.184	-0.02 (-0.11, 0.08)	0.701	0.07 (-0.03, 0.18)	0.187	0.148
ΣPCBs	-0.02 (-0.14, 0.11)	0.758	-0.09 (-0.23, 0.04)	0.182	0.397	-0.03 (-0.16, 0.09)	0.602	-0.06 (-0.20, 0.09)	0.432	0.791
ind-PCBs	-0.00 (-0.13, 0.12)	0.996	-0.06 (-0.20, 0.07)	0.354	0.449	-0.01 (-0.14, 0.11)	0.831	-0.03 (-0.17, 0.11)	0.702	0.873
DEHP	0.04 (-0.07, 0.15)	0.460	0.03 (-0.08, 0.15)	0.561	0.928	0.08 (-0.03, 0.19)	0.168	-0.01 (-0.13, 0.10)	0.808	0.263
HMW	0.06 (-0.05, 0.17)	0.262	0.04 (-0.07, 0.15)	0.500	0.787	0.09 (-0.02, 0.20)	0.101	-0.00 (-0.12, 0.11)	0.964	0.252
LMW	0.02 (-0.08, 0.12)	0.639	**0.18 (0.05, 0.30)**	**0.004**	0.058	-0.01 (-0.11, 0.09)	0.892	**0.16 (0.03, 0.28)**	**0.013**	**0.043**
DEs	-0.05 (-0.13, 0.03)	0.200	0.03 (-0.05, 0.11)	0.420	0.144	-0.04 (-0.12, 0.05)	0.391	0.05 (-0.02, 0.13)	0.178	0.121
DMs	0.02 (-0.04, 0.09)	0.479	0.03 (-0.05, 0.10)	0.500	0.966	0.02 (-0.05, 0.09)	0.653	-0.02 (-0.10, 0.06)	0.658	0.526
DAPs	-0.01 (-0.08, 0.07)	0.892	0.05 (-0.04, 0.14)	0.243	0.332	-0.01 (-0.09, 0.08)	0.850	0.02 (-0.07, 0.12)	0.605	0.607
**Externalizing symptoms (z-score)**										
HCB	-0.04 (-0.16, 0.09)	0.564	**0.20 (0.01, 0.39)**	**0.043**	**0.030**	-0.03 (-0.16, 0.10)	0.644	**0.22 (0.05, 0.40)**	**0.013**	**0.015**
DDT	-0.02 (-0.10, 0.07)	0.691	0.07 (-0.04, 0.18)	0.236	0.238	-0.03 (-0.11, 0.06)	0.570	0.10 (-0.02, 0.21)	0.117	0.114
DDE	-0.05 (-0.14, 0.05)	0.338	**0.17 (0.06, 0.27)**	**0.002**	**0.001**	-0.06 (-0.15, 0.04)	0.233	**0.14 (0.04, 0.25)**	**0.010**	**0.001**
ΣPCBs	-0.03 (-0.16, 0.10)	0.635	0.03 (-0.10, 0.17)	0.630	0.458	-0.03 (-0.16, 0.11)	0.690	0.04 (-0.11, 0.19)	0.597	0.472
ind-PCBs	-0.03 (-0.15, 0.10)	0.702	0.06 (-0.07, 0.20)	0.371	0.304	-0.02 (-0.15, 0.11)	0.784	0.07 (-0.07, 0.22)	0.325	0.308
DEHP	-0.01 (-0.12, 0.10)	0.895	0.08 (-0.04, 0.20)	0.175	0.285	0.07 (-0.05, 0.18)	0.273	0.06 (-0.07, 0.18)	0.362	0.929
HMW	-0.01 (-0.12, 0.09)	0.795	0.08 (-0.04, 0.20)	0.187	0.255	0.05 (-0.07, 0.16)	0.426	0.07 (-0.06, 0.19)	0.314	0.844
LMW	-0.06 (-0.16, 0.04)	0.252	**0.20 (0.07, 0.32)**	**0.002**	**0.002**	-0.08 (-0.19, 0.02)	0.126	**0.20 (0.07, 0.33)**	**0.003**	**0.001**
DEs	-0.07 (-0.15, 0.00)	0.062	**0.12 (0.03, 0.20)**	**0.006**	**0.001**	-0.06 (-0.15, 0.03)	0.170	**0.12 (0.04, 0.20)**	**0.005**	**0.004**
DMs	-0.02 (-0.09, 0.04)	0.468	**0.08 (0.00, 0.16)**	**0.037**	**0.039**	-0.05 (-0.13, 0.02)	0.157	0.07 (-0.02, 0.16)	0.133	**0.038**
DAPs	-0.05 (-0.13, 0.03)	0.214	**0.14 (0.05, 0.23)**	**0.002**	**0.002**	-0.07 (-0.16, 0.02)	0.112	**0.13 (0.03, 0.22)**	**0.011**	**0.003**
**ADHD symptoms (z-score)**										
HCB	-0.10 (-0.22, 0.02)	0.102	0.09 (-0.08, 0.26)	0.297	0.051	-0.10 (-0.23, 0.03)	0.142	0.14 (-0.02, 0.30)	0.095	**0.016**
DDT	-0.01 (-0.09, 0.07)	0.874	0.02 (-0.09, 0.13)	0.727	0.709	0.01 (-0.07, 0.10)	0.753	0.03 (-0.09, 0.14)	0.658	0.869
DDE	-0.05 (-0.14, 0.04)	0.251	0.07 (-0.03, 0.17)	0.157	**0.035**	-0.06 (-0.15, 0.04)	0.249	0.05 (-0.05, 0.16)	0.333	0.083
ΣPCBs	0.07 (-0.05, 0.19)	0.257	0.04 (-0.10, 0.17)	0.591	0.678	0.08 (-0.05, 0.21)	0.212	0.07 (-0.08, 0.21)	0.381	0.851
ind-PCBs	0.07 (-0.06, 0.19)	0.281	0.05 (-0.08, 0.18)	0.457	0.824	0.07 (-0.06, 0.20)	0.271	0.08 (-0.07, 0.22)	0.297	0.963

DEHP	0.03 (-0.07, 0.13)	0.531	0.04 (-0.07, 0.14)	0.486	0.944	**0.12 (0.01, 0.23)**	**0.031**	0.00 (-0.11, 0.12)	0.935	0.155
HMW	0.02 (-0.08, 0.12)	0.713	0.03 (-0.07, 0.14)	0.556	0.858	0.10 (-0.01, 0.20)	0.078	0.00 (-0.11, 0.12)	0.936	0.256
LMW	-0.08 (-0.17, 0.01)	0.101	**0.17 (0.06, 0.29)**	**0.002**	**0.001**	**-0.11 (-0.20, -0.01)**	**0.026**	**0.19 (0.07, 0.31)**	**0.001**	**<0.001**
DEs	-0.06 (-0.13, 0.01)	0.104	0.06 (-0.02, 0.13)	0.151	**0.032**	-0.05 (-0.13, 0.03)	0.229	**0.10 (0.02, 0.18)**	**0.012**	**0.009**
DMs	-0.02 (-0.08, 0.04)	0.545	**0.07 (0.00, 0.14)**	**0.036**	**0.046**	-0.04 (-0.11, 0.03)	0.295	0.06 (-0.02, 0.14)	0.147	0.072
DAPs	-0.04 (-0.11, 0.03)	0.257	**0.10 (0.02, 0.17)**	**0.019**	**0.012**	-0.06 (-0.14, 0.03)	0.177	**0.10 (0.02, 0.19)**	**0.022**	**0.009**

Internalizing, externalizing and ADHD symptoms are expressed as z-scores. All exposures are $\log_2$ transformed.

Values were derived from generalized estimating equations (GEE) analyses with child measures at 4, 6, 11 and 15 years.

Models were adjusted for child sex, exact age at assessment, child lipids (cholesterol and triglycerides, only for models with OCPs and PCBs), maternal age at delivery, maternal education, parity, household income, child BMI at 4 years, passive smoking at 4 years, breastfeeding duration, and place of residence. Models also included a multiplicative interaction term between exposure and child sex (male, female). The p-interaction values correspond to the exposure-sex interaction term.

[a] N=29 children with learning disabilities or ADHD diagnosis were excluded.

[b] N=48 preterm born children were excluded.

Bold font indicates $p < 0.05$.
